# Clotting events among hospitalized patients infected with COVID-19 in a large multisite cohort in the United States

**DOI:** 10.1371/journal.pone.0262352

**Published:** 2022-01-05

**Authors:** Sondra Maureen Nemetski, Andrew Ip, Joshua Josephs, Mira Hellmann

**Affiliations:** 1 Division of Pediatric Emergency Medicine, Department of Emergency Medicine, Hackensack University Medical Center—Joseph M. Sanzari Children’s Hospital, Hackensack Meridian Health, Hackensack, NJ, United States of America; 2 Department of Pediatrics, Hackensack Meridian School of Medicine, Nutley, NJ, United States of America; 3 Department of Hematology and Oncology, John Theurer Cancer Center, Hackensack University Medical Center, Hackensack Meridian Health, Hackensack, NJ, United States of America; 4 Department of Medicine, Hackensack Meridian School of Medicine, Nutley, NJ, United States of America; 5 Division of Hospital Medicine, Department of Medicine, Hackensack University Medical Center, Hackensack Meridian Health, Hackensack, NJ, United States of America; 6 Department of Obstetrics and Gynecology, Hackensack University Medical Center, Hackensack Meridian Health, Hackensack, NJ, United States of America; 7 Division of Gynecologic Oncology, John Theurer Cancer Center, Hackensack University Medical Center, Hackensack Meridian Health, Hackensack, NJ, United States of America; 8 Department of Oncology, Hackensack Meridian School of Medicine, Nutley, NJ, United States of America; Konkuk University, REPUBLIC OF KOREA

## Abstract

**Introduction:**

COVID-19 infection has been hypothesized to precipitate venous and arterial clotting events more frequently than other illnesses.

**Materials and methods:**

We demonstrate this increased risk of blood clots by comparing rates of venous and arterial clotting events in 4400 hospitalized COVID-19 patients in a large multisite clinical network in the United States examined from April through June of 2020, to patients hospitalized for non-COVID illness and influenza during the same time period and in 2019.

**Results:**

We demonstrate that COVID-19 increases the risk of venous thrombosis by two-fold compared to the general inpatient population and compared to people with influenza infection. Arterial and venous thrombosis were both common occurrences among patients with COVID-19 infection. Risk factors for thrombosis included male gender, older age, and diabetes. Patients with venous or arterial thrombosis had high rates of admission to the ICU, re-admission to the hospital, and death.

**Conclusion:**

Given the ongoing scientific discussion about the impact of clotting on COVID-19 disease progression, these results highlight the need to further elucidate the role of anticoagulation in COVID-19 patients, particularly outside the intensive care unit setting. Additionally, concerns regarding clotting and COVID-19 vaccines highlight the importance of addressing the alarmingly high rate of clotting events during actual COVID-19 infection when weighing the risks and benefits of vaccination.

## Introduction

As of September 12th, 2021, the COVID-19 pandemic has claimed the lives of over 4.5 million people, and caused significant morbidity worldwide [1]. Our understanding of this disease has increased substantially since it first emerged in December 2019, and it is now clear that both acute infection and a hyperimmune response to infection lead to multi-organ system effects. Elucidating these effects is crucial for the development of novel therapeutics and effective treatment protocols.

While much regarding the pathophysiology of this novel illness remains unknown, a growing body of evidence suggests that infection with SARS-CoV-2 precipitates a coagulopathy and that thromboses—both venous and arterial—contribute to morbidity and mortality [2–4]. Numerous case reports and retrospective chart reviews have reported incidences of thromboses in hospitalized COVID-19 patients, ranging from 2 to 79% for venous thromboses or thromboembolism (VT/VTE) [5–25] and 1.4 to 3.6% for arterial thromboses and embolic events (AT/ATE) [5,17,18,20,26,27]. Several studies have shown high rates of thrombosis despite anticoagulation [9–11,14–16,18–20,25]. To date, most of these studies have been limited by small sample sizes, and by a lack of comparative data for patients with non-COVID illness.

A hospital in New York City reported a rate of 1.7% of imaging-confirmed VTE among 921 COVID-19 patients admitted during the city’s first wave of the pandemic, as well as 11 patients with ischemic stroke and 2 with limb ischemia [17]. An observational study by Elbadawi, *et al* found acute thrombotic events, including VTE, acute ischemic stroke, and acute myocardial infarction (MI), in 5.5% of 892 hospitalized COVID-19 patients and 8.8% of 296 COVID-19 patients admitted to the ICU. Thrombosis was associated with higher mortality and increased need for intensive respiratory and hemodynamic support [5]. Al-Samkari, *et al* reported a thrombotic complication rate of 9.5% among 400 hospitalized patients, despite prophylactic anticoagulation. This included 4.7% of 256 noncritically ill patients and 18.1% of 144 critically ill patients for whom endotracheal intubation and mechanical ventilation was clinically indicated [6]. In the largest study to date examining rates of thromboembolic events in COVID-19 patients, Benito *et al* found an incidence for pulmonary embolism of 2.6% among 1275 patients admitted to a hospital in Barcelona in the Spring of 2020 [25]. While robust, these studies were limited by a lack of comparison to thrombotic events in patients hospitalized for non-COVID illness.

Here we present a retrospective analysis of a large database of COVID-19 patients admitted to a large healthcare network in New Jersey, an epicenter of COVID-19 infection during the initial “surge” in the United States between March and June 2020. We report the incidence of both venous and arterial thrombotic events in a cohort of 4451 COVID-19 patients in comparison to the rate of thrombotic events in non-COVID-19 patients admitted to the hospital during a similar time period in 2019, as well as patients hospitalized for influenza in 2019 or 2020. We also analyze the association between thrombotic events and morbidity—including admission to the ICU—and all cause mortality for these cohorts. Finally, we present the incidence of thrombotic events in COVID-19 patients who were on outpatient anticoagulation regimens prior to their Covid illness.

## Material and methods

### Patient population

This retrospective, observational, multicenter cohort study within the Hackensack Meridian Health (HMH) network utilized electronic health record (EHR)-derived data of COVID-19 patients who were hospitalized. Our primary objective was to compare the rates of clotting among hospitalized patients with COVID-19 versus patients admitted during the same time period for non-COVID illness or for influenza prior to the COVID-19 pandemic.

Database inclusion criteria for this study included 1) positive SARS-CoV-2 diagnosis by polymerase chain reaction and 2) hospitalization at one of HMH’s 13 hospitals between March 1, 2020 and June 29th, 2020. Pregnancy was the only exclusion criteria.

Hackensack Meridian Health Institutional Review Board (HMH IRB) approval was obtained for access to the prospective observational database, registered on clinicaltrials.gov (NCT04347993). The requirement for patient informed consent was waived by the IRB as this project represented a non-interventional study utilizing routinely collected data for secondary research purposes.

### Data sources and collection methodology

We collected data from HMH’s EHR which is utilized throughout the network. Hospitalized patients were flagged by the EHR if SARS-CoV-2 polymerase chain reaction tests were positive. EHR-generated reports served as our eligible cohort sample. Demographics, clinical characteristics, treatments, and outcomes were manually abstracted by research nurses and physicians from the John Theurer Cancer Center at HMH. Assignment of patients to our data team occurred in real-time but was not randomized. Data abstracted by the team were entered utilizing Research Electronic Data Capture (REDCap). Billing and collections data captured from HMH Business Intelligence was used to compare COVID-19 clotting events in 2020 to prior year data. ICD 10 codes of I21, I26, I63, I74, I80, I81, I82, O22, O88 were used to identify clotting events within the billing data source. A random selection of the billing data was compared to the REDCap data to ensure that all events were captured.

Demographic information was collected by an electronic face-sheet. Gender, race, or ethnicity was self-reported. ICU level support included all patients receiving mechanical ventilator support, patients hospitalized within a dedicated ICU, and patients with assignment to the ICU staff regardless of geographic placement (overflow during pandemic conditions). If multiple positive or indeterminate results were found in a patient’s record for SARS-CoV-2, the first initial positive test was used as the date of diagnosis.

### Statistical analysis

Statistical analysis was conducted using Stata® 12 (College Station, TX). Differences between groups were analyzed with t-tests or where more than two levels of the group existed with chi-squared testing with an alpha level of 0.05 pre-specified as being statistically significant. Risk ratios were calculated for the comparison between COVID and non-COVID patients and between COVID patients and those infected with influenza in 2019 and 2020.

## Results

### Baseline characteristics

4,451 patients were abstracted into the main database as described in the methods. Baseline characteristics were compared between patients with or without clotting events in the ICU or non-ICU setting. Several baseline factors, as shown in Table 1, appear to be associated with higher risk of clotting events. The median age of patients with clotting events was 66 vs 60 for patients without clotting events in both the non-ICU and ICU settings (p<0.05 for each). There were more male patients with clots in both cohorts (65% vs 57% in the non-ICU setting and 66% vs 57% in the ICU, p<0.05 for both). Diabetes also appeared to be a risk factor for increased clotting risk in both the non-ICU and ICU setting (33% vs 28%, p<0.05, and 42 vs 27%, p<0.05, respectively). Hypertension was a risk for increased clotting only in the ICU setting (61% vs 50%, p<0.05).

**Table 1:** Baseline characteristics of COVID-19 patients with and without clotting events (venous or arterial) in the non-ICU and ICU settings.

	Non-ICU		ICU	
	Clot	No Clot	Clot	No Clot
Mean Age (SD)	66* (16.1)	60 (18.2)	66* (14.1)	60 (20.3)
Men, %	65*	57	66*	57
Diabetes, %	33*	28	42*	27
Hypertension, %	51	54	61*	50
Coronary artery disease, %	14	16	13	15
Cancer, %	13	15	12	12
History of DVT, %	1	0	0	0
Rheumatologic disease, %	3	1	3	3
Current smoker, %	4	4	31*	40

*p-value < 0.05 for the difference between patients in each setting (non-ICU or ICU) who had clotting events vs the patients who did not have clotting events, as determined by the chi-squared test.

### Venous thrombotic events

**Fig 1:**
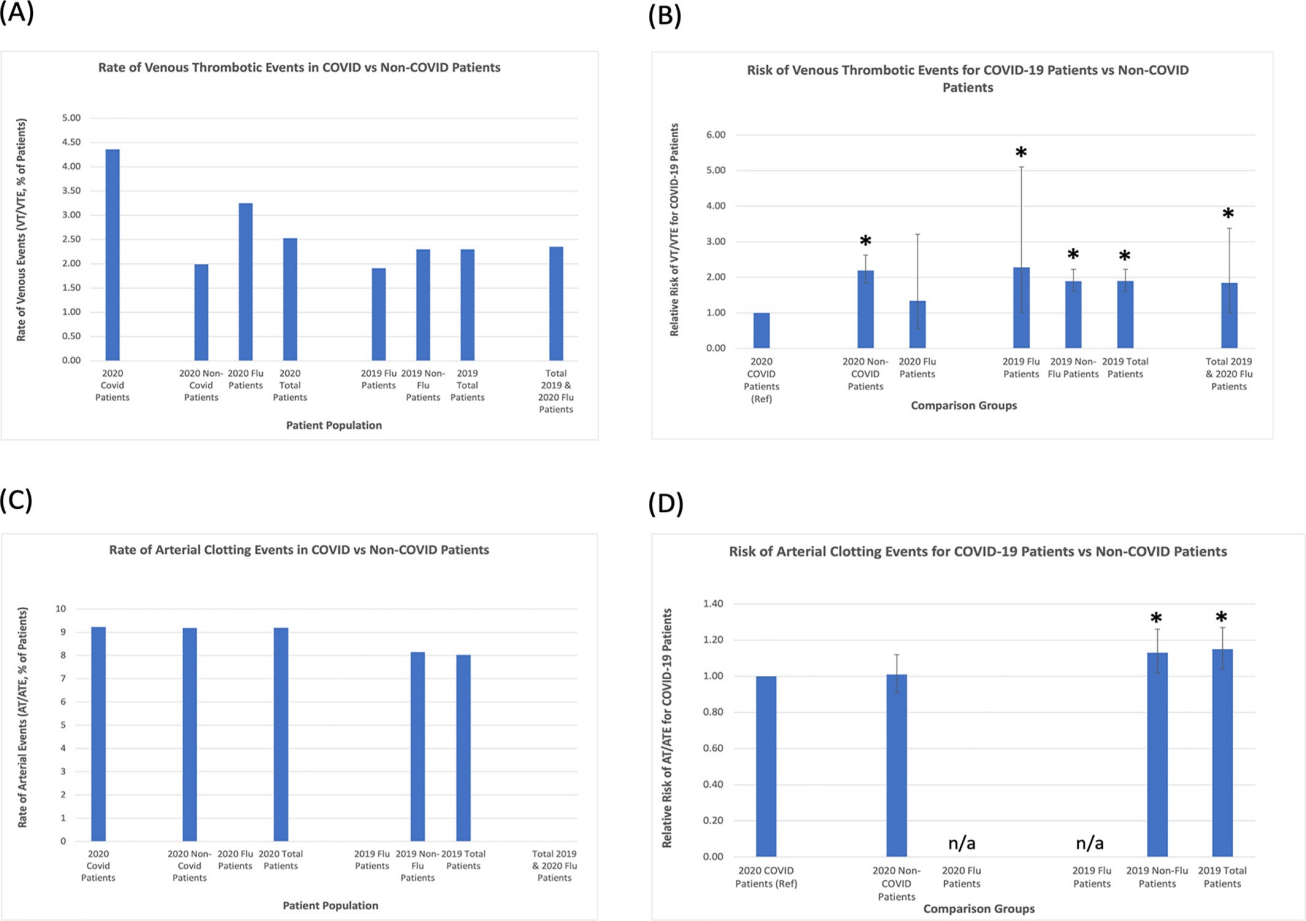
Rates and risk ratios of venous and arterial thrombosis in COVID-19 vs non-COVID patients. (A) Our cohort of hospitalized COVID-19 positive patients had higher rates of venous thrombotic events than non-COVID patients admitted to the hospital during the same time period in 2020 and historical controls admitted in 2019. (B) Patients admitted with COVID-19 had an increased risk of venous thromboses of 219% (RR 2.19, 95% CI 1.84–2.62, p < 0.0001) vs non-COVID patients admitted during the same time period in 2020, as well as an increased risk of 190% (RR1.90, 95% CI 1.61–2.23, p<0.0001) vs historical controls admitted during the same time period in 2019. Overall, COVID-19 patients had approximately twice the risk of clotting compared to influenza infected patients admitted in 2019 or 2020 (RR 1.85, 95% CI 1.02–3.38, p<0.05). (C) Rate of arterial thrombotic events in COVID-19 patients were similar to those in non-COVID patients admitted during the same time period in 2020, but higher than patients admitted during the same time period in 2019. (D) COVID-19 patients had an increased risk for arterial clots of 113% (RR 1.13, 95% CI 1.02–1.26) compared to non-flu patients and 115% (RR 1.15, 95% CI 1.04–1.27) compared all patients admitted in 2019. RR compared to influenza patients in 2019 and 2020 could not be calculated, as there were no arterial clots noted among patients admitted with influenza in either year. Bars represent 95% confidence intervals. *p-value statistically significant at less than 0.05 level. (VT/VTE = Venous Thrombosis/Venous Thromboembolism; AT/ATE = Arterial Thrombosis/Arterial Thromboembolism; RR = relative risk).

We compared rates of clotting in our COVID-19 database to historical controls using EHR generated reports from HMH, as described in our methods, first analyzing venous clotting events. As described in Table 2 and Fig 1A and 1B, 194 of the 4,451 COVID-19 patients in our cohort (4.36%) had a venous thrombotic or thromboembolic event (VT/VTE) vs 295 of 14,854 (1.99%) non-COVID patients hospitalized during the same time period in 2020, representing an increased risk of 219% (RR 2.19, 95% CI 1.84–2.62, p < 0.0001). We also analyzed all hospitalized patients from the same time period in 2019, and discovered 494 of 21,517 patients had VT/VTE events (2.30%); when compared to this cohort, the risk ratio for our COVID-19 patients is 1.90 (95% CI 1.61–2.23, p<0.0001). The rate of VT/VTE in influenza patients from 2019 and 2020 was also comparable (11/468 patients, or 2.35%) to non-COVID-19 hospitalized patients in 2019 and 2020. Overall, COVID-19 patients had approximately twice the risk of clotting compared to influenza infected patients (risk ratio 1.85, 95% CI 1.02–3.38, p<0.05).

**Table 2:** Rates and risk ratios of venous and arterial thrombosis in COVID-19 vs non-COVID patients.

	Sample Size	Venous Events (VT/VTE)	Rate of VT/VTE	Relative Risk of VT/VTE for COVID-19 patients vs Comparison Group (95% CI)	Arterial Events (AT/ATE, MI, Stroke)	Rate of AT/ATE	Relative Risk of AT/ATE for COVID-19 patients vs Comparison Group (95% CI)
**2020**							
-- Covid patients	4451	194	4.36%	**1.0**	411	9.23%	**1.0 (REF)**
-- non- Covid patients	14854	295	1.99%	2.19* (1.84–2.62)	1363	9.18%	**1.01 (0.91–1.12)**
-- Flu	154	5	3.25%	**1.34 (0.56–3.21)**	0	0%	**n/a**
-- Total patients	19310	489	2.53%	**-------------------**	1774	9.19%	**-----------------**
**2019**							
-- Flu pts	314	6	1.91%	2.28* (1.02–5.10)	0	0%	**n/a**
-- non-Flu patients	21203	488	2.30%	1.89* (1.61–2.23)	1727	8.15%	1.13* (1.02–1.26)
-- Total patients	21517	494	2.30%	1.90* (1.61–2.23)	1727	8.03%	1.15* (1.04–1.27)
Total 2019 & 2020 Flu patients	468	11	2.35%	1.85* (1.02–3.38)	0	0%	**n/a**

*p-value statistically significant at less than 0.05 level. (VT/VTE = Venous Thrombosis/Venous Thromboembolism; AT/ATE = Arterial Thrombosis/Arterial Thromboembolism; MI = Myocardial Infarction).

### Arterial clotting events

Next, we analyzed the rates of arterial clotting events, such as myocardial infarction, stroke, and arterial thrombosis, also shown in Table 2 and in Fig 1C and 1D. We found an increased number of arterial events as compared to venous events, with 411 arterial clotting events in 4,451 COVID-19 patients (9.23%). Of these, 158 were coded as NSTEMI type 2 demand ischemia. When compared to in 2020 non-COVID-19 hospitalized patients, there were no statistically significant differences found; however, rates of AT/ATE in COVID-19 hospitalizations were statistically significantly higher than the rate of arterial events in patients hospitalized in 2019. Notably, in contrast to our COVID-19 population, there were no arterial clotting events documented for patients hospitalized with other influenza-like illnesses in 2019 or 2020 (Table 2 and Fig 1C).

### Clotting events and morbidity and mortality

**Fig 2:**
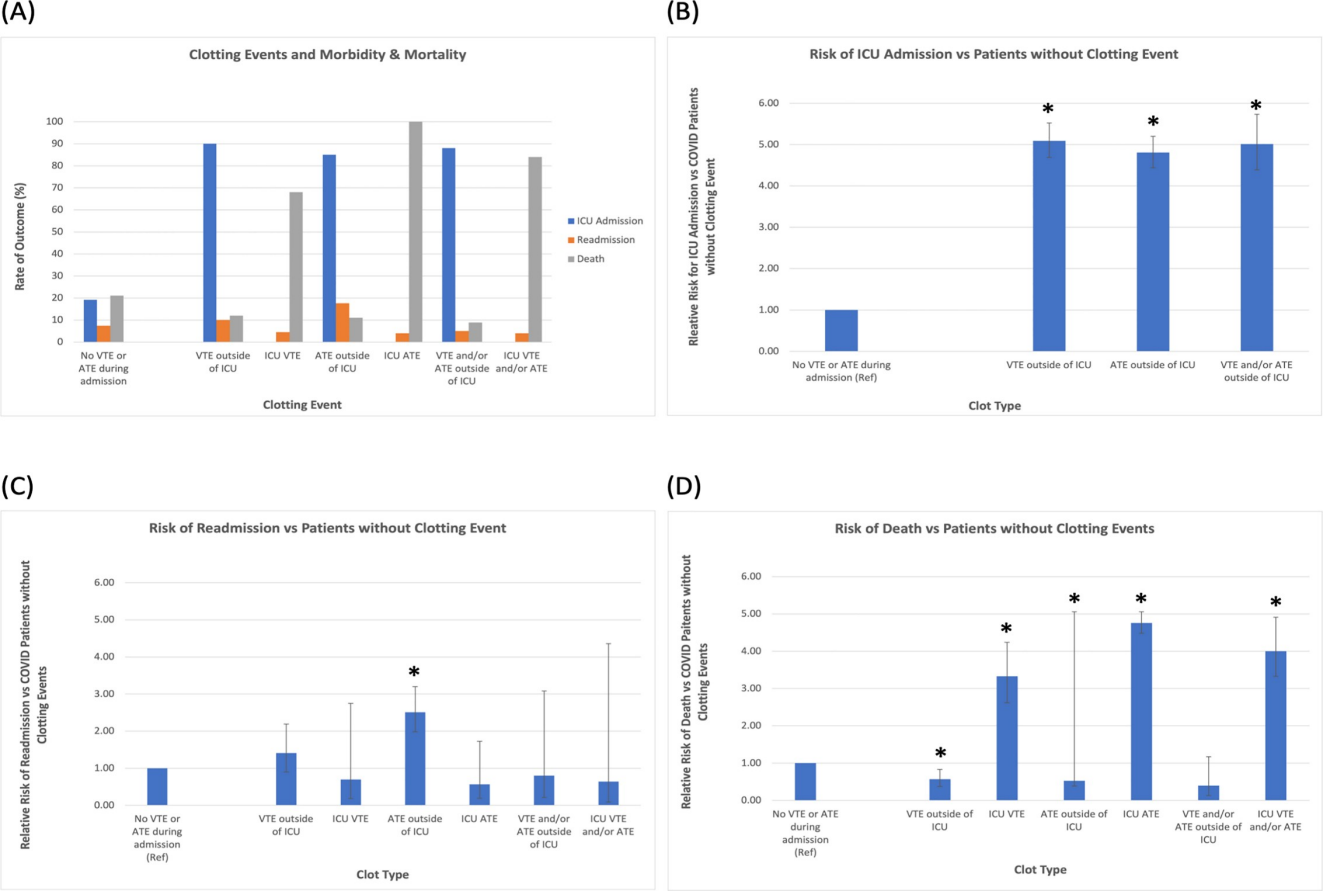
Impact of clotting events on risk of ICU admission, re-admission, and death. (A) Rates of ICU admission, readmission to the hospital after discharge, and death for hospitalized COVID-19 patients with and without venous or arterial clots during their initial COVID admission. (B) COVID-19 patients who developed venous and/or arterial clots during admission were significantly more likely to require escalation to ICU care than their counterparts who did have clotting events. (C) COVID-19 patients who developed arterial clots outside of the ICU were significantly more likely to be readmitted to the hospital. While the risks of readmission for other COVID-19 patients with thrombotic events seem lower, this is likely skewed by the fact that many of those patients did not survive to initial hospital discharge, as illustrated by (D) which shows the significantly higher risk of death among COVID-19 with clotting events than those without. In the small number of patients with clots who were not admitted to the ICU, mortality was less than in the general hospital population; however, this is probably biased by the large number of palliative and hospice care patients who passed away on the floor and would not have been admitted to the ICU. Bars represent 95% confidence intervals. *p-value statistically significant at less than 0.05 level. See Table 3 for RR values. (VT/VTE = Venous Thrombosis/Venous Thromboembolism; AT/ATE = Arterial Thrombosis/Arterial Thromboembolism; RR = Relative Risk).

In patients with either VT/VTE, AT/ATE, or both, risk of admission to the ICU was in excess of 80 percent. Risks of hospital readmission or ICU admission were 2–5 times higher in patients who had clotting events than those who did not (Table 3 and Fig 2). In the small number of patients with clots who were not admitted to the ICU, mortality was less than in the general hospital population; however, this is probably biased by the large number of palliative and hospice care patients who passed away on the floor and would not have been admitted to the ICU.

**Table 3:** Rates and risk ratios of ICU admission, readmission to the hospital, and death among COVID-19 patients with clotting events.

	Rate of ICU Admission	Relative Risk of ICU Admission vs Patients without Clotting Event (95% CI)	Rate of Readmission	Relative Risk of Readmission vs Patients without Clotting (95% CI)	Rate of Death	Risk of Death vs Patients Without Clotting Event (95% CI)
VTE outside of ICU	90%	5.09* (4.69–5.52)	10%	**1.41 (0.90–2.19)**	12%	0.57* (0.38–0.83)
ICU VTE	--------------	**----------------**	4.5%	**0.70 (0.18–2.75)**	68%	3.33* (2.62–4.24)
ATE outside of ICU	85%	4.81* (4.44–5.20)	17.6%	2.51* (1.98–3.20)	11%	0.53* (0.39–0.69)
ICU ATE	--------------	**----------------**	4%	**0.57 (0.19–1.73)**	100%	4.76* (4.48–5.06)
VTE and/or ATE outside of ICU	88%	5.01* (4.39–5.73)	5%	**0.80 (0.21–3.08)**	8.9%	**0.40 (0.13–1.17)**
ICU VTE and/or ATE	----------------	**----------------**	4%	**0.64 (0.09–4.36)**	84%	4.0* (3.33–4.91)
No VTE or ATE during admission	19.2%	**1.0 (Ref)**	7.4%	**1.0 (Ref)**	21%	**1.0 (Ref)**

*p-value statistically significant at less than 0.05 level. (VT/VTE = Venous Thrombosis/Venous Thromboembolism; AT/ATE = Arterial Thrombosis/Arterial Thromboembolism).

### Prior anticoagulation and clotting risk before ICU admission

Lastly, we analyzed if the risk of blood clots differed amongst those who were receiving anticoagulation prior to hospitalization vs those were not (Table 4). We found no statistically significant difference, although there appeared to be a trend towards less risk of clotting events for the cohort on anticoagulation prior to hospitalization. For example, only 1.7% of patients on anticoagulation prior to hospitalization had venous clotting events, as compared to 4.6% of patients who had no exposure to anticoagulation prior to COVID hospitalization (p = 0.44). A similar trend was seen in the subpopulation with arterial clotting events. Because a majority of patients received at least prophylactic anticoagulation in the hospital, generally even before transfer to the ICU, we did not examine pre-hospitalization anticoagulation and ICU clotting events.

**Table 4:** Risk ratios (95% CI) of clotting events in COVID-19 patients based on the use of anti-coagulation prior to admission.

	Pre-Hospital AC	No Pre-hospital AC
VT/VTE outside of ICU	1.0 (Ref)	2.0 (0.50–7.94)
ICU VT/VTE	N/A	N/A
AT/ATE outside of ICU	1.0 (Ref)	3.5 (0.90–14.0)
VT/VTE and AT/ATE outside of ICU	1.0 (Ref)	0.37 (0.09–1.52)

(VT/VTE = Venous Thrombosis/Venous Thromboembolism; AT/ATE = Arterial Thrombosis/Arterial Thromboembolism; AC = Anticoagulation).

### Coagulation parameters and clotting

D-dimer levels drawn within 48 hours of presentation to the hospital were available for 1572 patients, of whom 1172 were initially admitted to the general hospital floors and 400 were initially admitted to the ICU. For both cohorts, we observed no significant difference when comparing the initial D-dimer levels between patients who did or did not develop venous or arterial clots during their admission (Tables 5 and 6).

**Table 5:** Comparison of D-dimer levels in the first 48 hours of hospitalization between those with and without thrombosis admitted to the general hospital floors.

N = 1172	Overall	Patients without Clot	Patients with Clot	P-value for Difference*
Median D-dimer	1.04	1.05	0.97	-----------------
Mean D-dimer	3.43	3.38	3.80	0.70
Standard Deviation	9.34	9.00	11.80	-----------------

*The test performed here is the two sample T-test with unequal variances. If we instead use a non-parametric test to account for the fact that the distributions are likely non normal then we get a Kolomogorov Smirnov p-value of 0.25, which suggests no difference between the groups. (D-dimer levels reported in mcg/mL).

**Table 6:** Comparison of D-dimer levels in the first 48 hours of hospitalization between those with and without thrombosis who were admitted to the ICU.

N = 400	Overall	Patients without Clot	Patients with Clot	P-value for Difference
Median D-dimer	2.39	2.31	2.91	-----------------
Mean D-dimer	7.47	6.42	9.37	0.11
Standard Deviation	15.62	12.00	20.59	-----------------

*The test performed here is the two sample T-test with unequal variances. If we instead use a non-parametric test to account for the fact that the distributions are likely non normal then we get a Kolomogorov Smirnov p-value of 0.21, which suggests no difference between the groups. (D-dimer levels reported in mcg/mL).

## Discussion

The SARS-Cov-2 novel coronavirus has infected over 40 million patients with over 650,000 deaths documented in the United States to date [28]. Several characteristics are currently established to be risk factors for severe disease and mortality, such as male gender, age over 60, hypertension, and obesity.

Thromboembolic disease emerged early in the pandemic to be a contributing factor to overall morbidity and mortality in affected patients. Estimates of thrombotic disease vary widely, with previous studies ranging from small case series to multi-center prospective studies [5–27]. Prior to this publication, the largest cohort examined included 1275 patients at a single center [25].

Our study looks at the largest database to date, with 4451 PCR positive COVID-19 patients admitted to the Hackensack Meridian Healthcare system during the first surge of COVID-19 cases in the Northeastern United States. Analysis of the rate of VT/VTE in the COVID-19 infected population (4.36%) demonstrated a statistically significant increase when compared to non-COVID-19 patients for 2020 (1.99%) as well as patients admitted in the same timeframe in 2019 (2.30%). In addition, we found the rate of VT/VTE in patients admitted with influenza in 2019 (1.91%) was comparable to the rate of VTE in non-COVID patients hospitalized during 2019 and 2020.

We also analyzed the rate of arterial thrombotic events (AT/ATE) in COVID-19 patients, and found the rate to be higher than that of VT/VTE at 9.23%; furthermore, the rate of AT/ATE was higher than patients hospitalized in 2019 at a rate of 8.15% (RR 1.13, p<0.05). Furthermore, rates of admission to the ICU, readmission to the hospital, and death, were all increased in patients with thrombotic events in the COVID-19 patient population, with risk of death highest among patients who developed an arterial clot while admitted to the ICU (100%).

Several theories have postulated the mechanism for increased risk of thromboses in COVID-19 patients, including possible aberrant forms of disseminated intravascular coagulation (DIC) [29], contribution of cytokine storm [2,30], and thrombotic microangiopathy (TMA) [31], among others. The exact mechanism is still unclear. Our study demonstrates that outpatient anticoagulation does not decrease the risk of developing VTE, and other studies have demonstrated that VTE developed in patients who were on anticoagulation during their hospital stay [11], indicating that the underlying mechanism is different than the standard hypercoagulable pathways [32–34].

D-dimers levels are used as markers to indicate thrombotic and inflammatory events, and prior literature has demonstrated a correlation between elevated d-dimer levels and increased risk of venous thromboses and worse outcomes in COVID-19 patients [9,35] D-dimers levels were available for analysis in 1572 of our patients, 1172 of whom were admitted to the general wards, and 400 admitted to the ICU. We did not find a statistically significant difference in d-dimer levels between patients with or without a clot, but this may be due to the fact that our study was not powered to use d-dimers as an endpoint. Furthermore, during the study period, d-dimers were not uniformly collected for all COVID-19 patients, and only one third of our patients had d-dimers available, further limiting our statistical analysis.

Manifestation of COVID-19-associated thrombosis is quite variable, highlighting the increased risk of both venous and arterial thromboses. Reports have documented cerebrovascular accidents in otherwise young, healthy patients, significantly high rates of clotting in hemodialysis machines [24], and arterial thrombi to the digits (COVID toes) [36]. Pulmonary embolism appears to be the most common manifestation of thrombosis in this patient population.

It is clear that anticoagulation is an essential component in the management of patients with COVID-19, though the exact role is still unclear [30]. Inpatient prophylaxis does not appear to be protective enough, but full anticoagulation carries a significant risk of bleeding, though it has been associated with decreased mortality in patients requiring mechanical ventilation [37]. There are currently open studies underway exploring the most beneficial form of anticoagulation. Our study clearly demonstrates that emphasis should be placed on prevention of thrombosis to avoid admission to the ICU. Outcomes of patients with thrombosis admitted to the ICU are very poor, with the risk of death of 100% for patients admitted to the ICU with arterial thrombosis, and 84% for patients with venous or arterial thrombosis.

A recent meta-analysis analyzed 102 papers documenting the rate of VTE and ATE in COVID-19 patients. The total number of patients in the study amounted to 64,503, with a cumulative rate of VTE of 14.7% and ATE of 3.9%. The differences in rates may be due to the method of data analysis, due to the fact that at least 33% of the studies were in ICU patients with COVID-19 where the incidence of VTE is much higher, or due to the fact that institutions varied widely in how accessible imaging studies were available to document VTE [38].

Furthermore, and perhaps an alarming finding, is that similar to other studies [11,32], outpatient anticoagulation did not affect the rate of thrombotic events for patients hospitalized with COVID-19, though there was a non-significant trend towards decreased risk of thrombosis present.

It is important to highlight the significance of the increased rate of VTE/ATE in COVID-19 patients. In April 2020, Johnson and Johnson Janssen COVID-19 vaccine’s United States’ Emergency Use Authorization was temporarily suspended due to several thrombotic events that occurred post vaccination. This adverse outcome was initially documented in six patients, one of whom died. Approximately 6.8 million doses of the vaccine were administered at that time, making the incidence of this complication exceedingly rare. The suspension was subsequently lifted, with a warning. Comparing the rates of VTE in patients receiving the vaccine, the incidence is exponentially smaller than in patients with COVID-19.

Weaknesses of our study include the retrospective nature of the study, which may limit some analyses. Our data was collected from electronic medical records, and database information that was manually entered, which may be prone to human error. Furthermore, the period of time of the data collection was during the height of the initial disease spread in our area, with hospitals within the HMH system being overwhelmed. This may have resulted in limited access to radiologic studies, thereby underestimating the true number of thrombotic events, since many may have gone undiagnosed.

Strengths of our study include the largest database of COVID-19 patients analyzed for thrombotic events to date, resulting in the highest number of thrombotic phenomena in this patient population. Furthermore, data was obtained from a dedicated COVID-19 database, thereby likely reducing the amount of missing information. In addition, this is the largest series to include control groups–both concurrent controls (non-COVID patients in 2020) and historic controls (influenza patients in 2019). Most other studies have been observational studies without a control arm. Our lower rate of thrombotic events relative to other published numbers is likely due to the large number of patients in our study, and our statistically significant findings, indicating that our rates more likely reflect the real incidences of thromboses in COVID-19 patients.

It is important to note that our study, as well as most other studies published on this topic, encompass only the initial circulating strains of SARS-CoV-2. Detailed analyses of clotting events during infections with newer strains of the virus, including the Delta variant, have not yet been published. While anticoagulation remains standard practice for patients admitted to the hospital with severe COVID-19 illness, it is imperative that research into the risks and pathophysiology of clotting during COVID-19 illness, as well as studies comparing the efficacy of various anticoagulation protocols, continue as additional viral strains emerge.

## Conclusion

Thromboembolic disease, both venous and arterial, is significantly increased in patients with COVID-19, and is correlated with poorer outcomes. More studies are necessary to elicit the exact mechanism responsible for this phenomenon to allow for preventative measures to be implemented.

## Abbreviations

VT/VTE: Venous Thrombosis/Venous Thromboembolism
AT/ATE: Arterial Thrombosis/Arterial Thromboembolism
MI: myocardial infarction
HMH: Hackensack Meridian Health
EHR: Electronic Health Record

## References

[1] Johns Hopkins University & Medicine. COVID-19 Map. In: Johns Hopkins Coronavirus Resource Center [Internet]. [cited 12 Sep 2021]. Available: https://coronavirus.jhu.edu/map.html.

[2] Mackman N, Antoniak S, Wolberg AS, Kasthuri R, Key NS. Coagulation Abnormalities and Thrombosis in Patients Infected With SARS-CoV-2 and Other Pandemic Viruses. Arterioscler Thromb Vasc Biol. 2020;40: 2033–2044. doi: 10.1161/ATVBAHA.120.314514 32657623 PMC7447001

[3] Ahmed S, Zimba O, Gasparyan AY. Thrombosis in Coronavirus disease 2019 (COVID-19) through the prism of Virchow’s triad. Clin Rheumatol. 2020;39: 2529–2543. doi: 10.1007/s10067-020-05275-1 32654082 PMC7353835

[4] Frazer JS, Tyrynis Everden AJ. Emerging patterns of hypercoagulability associated with critical COVID-19: A review. Trends Anaesth Crit Care. 2020;34: 4–13. doi: 10.1016/j.tacc.2020.07.004 PMC7346831 38620391

[5] Ayman Elbadawi, Elgendy Islam Y., Sahai Aditya, Bhandari Rohan, McCarthy Meghann, Gomes Marcelo, et al. Incidence and Outcomes of Thrombotic Events in Symptomatic Patients With COVID-19. Arterioscler Thromb Vasc Biol. 0: ATVBAHA.120.315304. doi: 10.1161/ATVBAHA.120.315304 32990453 PMC7770038

[6] Al-Samkari H, Karp Leaf RS, Dzik WH, Carlson JCT, Fogerty AE, Waheed A, et al. COVID-19 and coagulation: bleeding and thrombotic manifestations of SARS-CoV-2 infection. Blood. 2020;136: 489–500. doi: 10.1182/blood.2020006520 32492712 PMC7378457

[7] Mei F, Fan J, Yuan J, Liang Z, Wang K, Sun J, et al. Comparison of Venous Thromboembolism Risks Between COVID-19 Pneumonia and Community-Acquired Pneumonia Patients. Arterioscler Thromb Vasc Biol. 2020;40: 2332–2337. doi: 10.1161/ATVBAHA.120.314779 32628040 PMC7446987

[8] Stoneham SM, Milne KM, Nuttall E, Frew GH, Sturrock BR, Sivaloganathan H, et al. Thrombotic risk in COVID-19: a case series and case–control study. Clin Med. 2020;20: e76–e81. doi: 10.7861/clinmed.2020-0228 32423903 PMC7385762

[9] Demelo-Rodríguez P, Cervilla-Muñoz E, Ordieres-Ortega L, Parra-Virto A, Toledano-Macías M, Toledo-Samaniego N, et al. Incidence of asymptomatic deep vein thrombosis in patients with COVID-19 pneumonia and elevated D-dimer levels. Thromb Res. 2020;192: 23–26. doi: 10.1016/j.thromres.2020.05.018 32405101 PMC7219400

[10] Longchamp A, Longchamp J, Manzocchi‐Besson S, Whiting L, Haller C, Jeanneret S, et al. Venous thromboembolism in critically Ill patients with COVID‐19: Results of a screening study for deep vein thrombosis. Cannegieter S, editor. Res Pract Thromb Haemost. 2020;4: 842–847. doi: 10.1002/rth2.12376 32685893 PMC7272794

[11] Middeldorp S, Coppens M, Haaps TF, Foppen M, Vlaar AP, Müller MCA, et al. Incidence of venous thromboembolism in hospitalized patients with COVID‐19. J Thromb Haemost. 2020;18: 1995–2002. doi: 10.1111/jth.14888 32369666 PMC7497052

[12] Nahum J, Morichau-Beauchant T, Daviaud F, Echegut P, Fichet J, Maillet J-M, et al. Venous Thrombosis Among Critically Ill Patients With Coronavirus Disease 2019 (COVID-19). JAMA Netw Open. 2020;3: e2010478. doi: 10.1001/jamanetworkopen.2020.10478 32469410 PMC7260620

[13] Hippensteel JA, Burnham EL, Jolley SE. Prevalence of venous thromboembolism in critically ill patients with COVID‐19. Br J Haematol. 2020;190. doi: 10.1111/bjh.17190 32484907 PMC7300700

[14] Chen S, Zhang D, Zheng T, Yu Y, Jiang J. DVT incidence and risk factors in critically ill patients with COVID-19. J Thromb Thrombolysis. 2020 [cited 5 Nov 2020]. doi: 10.1007/s11239-020-02181-w 32607652 PMC7324310

[15] Kaminetzky M, Moore W, Fansiwala K, Babb JS, Kaminetzky D, Horwitz LI, et al. Pulmonary Embolism on CTPA in COVID-19 Patients. Radiol Cardiothorac Imaging. 2020;2: e200308. doi: 10.1148/ryct.2020200308 33778610 PMC7336753

[16] Visser C, Sprenger RA, van den Bout HJ, Boer DP, el Moussaoui R, den Hollander JG. Venous thrombotic events in severe and critically COVID-19 patients despite high dose prophylactic low-molecular-weight heparin. Thromb Update. 2020;1: 100006. doi: 10.1016/j.tru.2020.100006 PMC7434635 38620665

[17] Hanif A, Khan S, Mantri N, Hanif S, Saleh M, Alla Y, et al. Thrombotic complications and anticoagulation in COVID-19 pneumonia: a New York City hospital experience. Ann Hematol. 2020;99: 2323–2328. doi: 10.1007/s00277-020-04216-x 32808105 PMC7430929

[18] Klok FA, Kruip MJHA, van der Meer NJM, Arbous MS, Gommers DAMPJ, Kant KM, et al. Incidence of thrombotic complications in critically ill ICU patients with COVID-19. Thromb Res. 2020;191: 145–147. doi: 10.1016/j.thromres.2020.04.013 32291094 PMC7146714

[19] Llitjos J, Leclerc M, Chochois C, Monsallier J, Ramakers M, Auvray M, et al. High incidence of venous thromboembolic events in anticoagulated severe COVID‐19 patients. J Thromb Haemost. 2020;18: 1743–1746. doi: 10.1111/jth.14869 32320517 PMC7264774

[20] Lodigiani C, Iapichino G, Carenzo L, Cecconi M, Ferrazzi P, Sebastian T, et al. Venous and arterial thromboembolic complications in COVID-19 patients admitted to an academic hospital in Milan, Italy. Thromb Res. 2020;191: 9–14. doi: 10.1016/j.thromres.2020.04.024 32353746 PMC7177070

[21] Julien Poissy, Julien Goutay, Morgan Caplan, Erika Parmentier, Thibault Duburcq, Fanny Lassalle, et al. Pulmonary Embolism in Patients With COVID-19. Circulation. 2020;142: 184–186. doi: 10.1161/CIRCULATIONAHA.120.047430 32330083

[22] Léonard-Lorant I, Delabranche X, Séverac F, Helms J, Pauzet C, Collange O, et al. Acute Pulmonary Embolism in Patients with COVID-19 at CT Angiography and Relationship to d-Dimer Levels. Radiology. 2020;296: E189–E191. doi: 10.1148/radiol.2020201561 32324102 PMC7233397

[23] Wichmann D, Sperhake J-P, Lütgehetmann M, Steurer S, Edler C, Heinemann A, et al. Autopsy Findings and Venous Thromboembolism in Patients With COVID-19. Ann Intern Med. 2020 [cited 4 Feb 2021]. doi: 10.7326/M20-2003 32374815 PMC7240772

[24] Helms J, Tacquard C, Severac F, Leonard-Lorant I, Ohana M, Delabranche X, et al. High risk of thrombosis in patients with severe SARS-CoV-2 infection: a multicenter prospective cohort study. Intensive Care Med. 2020; 1–10. doi: 10.1007/s00134-020-06062-x 32367170 PMC7197634

[25] Benito N, Filella D, Mateo J, Fortuna AM, Gutierrez-Alliende JE, Hernandez N, et al. Pulmonary Thrombosis or Embolism in a Large Cohort of Hospitalized Patients With Covid-19. Front Med. 2020;7: 557. doi: 10.3389/fmed.2020.00557 32984388 PMC7477312

[26] Fara MG, Stein LK, Skliut M, Morgello S, Fifi JT, Dhamoon MS. Macrothrombosis and stroke in patients with mild Covid‐19 infection. J Thromb Haemost. 2020;18: 2031–2033. doi: 10.1111/jth.14938 32464707 PMC7283879

[27] Singh B, Aly R, Kaur P, Gupta S, Vasudev R, Virk HS, et al. COVID-19 Infection and Arterial Thrombosis: Report of Three Cases. Ann Vasc Surg. 2020; S0890509620307962. doi: 10.1016/j.avsg.2020.08.115 32889160 PMC7462876

[28] CDC. COVID Data Tracker. In: Centers for Disease Control and Prevention [Internet]. [cited 12 Sep 2021]. Available: https://covid.cdc.gov/covid-data-tracker.

[29] Levi M, Thachil J, Iba T, Levy JH. Coagulation abnormalities and thrombosis in patients with COVID-19. Lancet Haematol. 2020;7: e438–e440. doi: 10.1016/S2352-3026(20)30145-9 32407672 PMC7213964

[30] Hanff TC, Mohareb AM, Giri J, Cohen JB, Chirinos JA. Thrombosis in COVID-19. Am J Hematol. 2020;95: 1578–1589. doi: 10.1002/ajh.25982 32857878 PMC7674272

[31] Merrill JT, Erkan D, Winakur J, James JA. Emerging evidence of a COVID-19 thrombotic syndrome has treatment implications. Nat Rev Rheumatol. 2020;16: 581–589. doi: 10.1038/s41584-020-0474-5 32733003 PMC7391481

[32] Griffin DO, Jensen A, Khan M, Chin J, Chin K, Parnell R, et al. Arterial thromboembolic complications in COVID-19 in low-risk patients despite prophylaxis. Br J Haematol. 2020;190: e11–e13. doi: 10.1111/bjh.16792 32374029 PMC7267572

[33] Connors JM, Levy JH. COVID-19 and its implications for thrombosis and anticoagulation. Blood. 2020;135: 2033–2040. doi: 10.1182/blood.2020006000 32339221 PMC7273827

[34] Giannis D, Ziogas IA, Gianni P. Coagulation disorders in coronavirus infected patients: COVID-19, SARS-CoV-1, MERS-CoV and lessons from the past. J Clin Virol. 2020;127: 104362. doi: 10.1016/j.jcv.2020.104362 32305883 PMC7195278

[35] Eljilany I, Elzouki A-N. D-Dimer, Fibrinogen, and IL-6 in COVID-19 Patients with Suspected Venous Thromboembolism: A Narrative Review. Vasc Health Risk Manag. 2020;16: 455–462. doi: 10.2147/VHRM.S280962 33223833 PMC7672709

[36] Zhou B, She J, Wang Y, Ma X. Venous thrombosis and arteriosclerosis obliterans of lower extremities in a very severe patient with 2019 novel coronavirus disease: a case report. J Thromb Thrombolysis. 2020;50: 229–232. doi: 10.1007/s11239-020-02084-w 32306290 PMC7165253

[37] Paranjpe I, Fuster V, Lala A, Russak AJ, Glicksberg BS, Levin MA, et al. Association of Treatment Dose Anticoagulation With In-Hospital Survival Among Hospitalized Patients With COVID-19. J Am Coll Cardiol. 2020;76: 122–124. doi: 10.1016/j.jacc.2020.05.001 32387623 PMC7202841

[38] Tan BK, Mainbourg S, Friggeri A, Bertoletti L, Douplat M, Dargaud Y, et al. Arterial and venous thromboembolism in COVID-19: a study-level meta-analysis. Thorax. 2021; thoraxjnl-2020-215383. doi: 10.1136/thoraxjnl-2020-215383 33622981

